# Associations and limited shared genetic aetiology between bipolar disorder and cardiometabolic traits in the UK Biobank

**DOI:** 10.1017/S0033291721000945

**Published:** 2022-12

**Authors:** Anna E. Fürtjes, Jonathan R. I. Coleman, Jess Tyrrell, Cathryn M. Lewis, Saskia P. Hagenaars

**Affiliations:** 1 Social, Genetic and Developmental Psychiatry Centre, Institute of Psychiatry, Psychology & Neuroscience, King's College London, London, UK; 2 National Institute for Health Research Maudsley Biomedical Research Centre, South London and Maudsley NHS Trust, London, UK; 3 Genetics of Complex Traits, The College of Medicine and Health, University of Exeter, The RILD Building, RD&E Hospital, Exeter, EX2 5DW, UK

**Keywords:** Bipolar disorder, cardiovascular disease, cardiometabolic abnormalities, biomarkers, polygenic score, genetic correlation

## Abstract

**Background:**

People with bipolar disorder (BPD) are more likely to die prematurely, which is partly attributed to comorbid cardiometabolic traits. Previous studies report cardiometabolic abnormalities in BPD, but their shared aetiology remains poorly understood. This study examined the phenotypic associations and shared genetic aetiology between BPD and various cardiometabolic traits.

**Methods:**

In a subset of the UK Biobank sample (*N* = 61 508) we investigated phenotypic associations between BPD (*n*_cases_ = 4186) and cardiometabolic traits, represented by biomarkers, anthropometric traits and cardiometabolic diseases. To determine shared genetic aetiology in European ancestry, polygenic risk scores (PRS) and genetic correlations were calculated between BPD and cardiometabolic traits.

**Results:**

Several traits were significantly associated with increased risk for BPD, namely low total cholesterol, low high-density lipoprotein cholesterol, high triglycerides, high glycated haemoglobin, low systolic blood pressure, high body mass index, high waist-to-hip ratio; and stroke, coronary artery disease and type 2 diabetes diagnosis. BPD was associated with higher polygenic risk for triglycerides, waist-to-hip ratio, coronary artery disease and type 2 diabetes. Shared genetic aetiology persisted for coronary artery disease, when correcting PRS associations for cardiometabolic base phenotypes. Associations were not replicated using genetic correlations.

**Conclusions:**

This large study identified increased phenotypic cardiometabolic abnormalities in BPD participants. It is found that the comorbidity of coronary artery disease may be based on shared genetic aetiology. These results motivate hypothesis-driven research to consider individual cardiometabolic traits rather than a composite metabolic syndrome when attempting to disentangle driving mechanisms of cardiometabolic abnormalities in BPD.

## Introduction

Bipolar disorder (BPD) is among the 20 most debilitating neuropsychiatric diseases (World Health Organization, 2008), and is often accompanied by excess morbidity and premature mortality (Crump, Sundquist, Winkleby, & Sundquist, 2013). This co-occurrence has been attributed to comorbid cardiovascular and metabolic diseases, including coronary artery disease, stroke and type 2 diabetes, as well as risk factors such as high blood lipid levels and body mass measures (Penninx & Lange, 2018).

Epidemiological meta-analyses established a phenotypic link between BPD and obesity, high blood pressure and abnormal lipid levels (relative risk of metabolic abnormalities = 1.58, 95% CI 1.24–2.03) (Vancampfort et al., 2015), as well as type 2 diabetes (relative risk = 1.98, 95% CI 1.6–2.4) (Vancampfort et al., 2016). Results for coronary artery disease and BPD have been inconsistent: meta-analysis found associations in longitudinal (adjusted hazard ratio = 1.57, 95% CI 1.28–1.93), but not cross-sectional data (Correll et al., 2017b). Some studies even support a bidirectional relationship, meaning that cardiometabolic traits increase the risk for BPD, and *vice versa* (Lopresti & Drummond, 2013). However, it is unclear whether cardiometabolic abnormalities are inherent properties of BPD, or if the comorbidities implicate environmental influences – such as medication and lifestyle – or genetics.

BPD and cardiometabolic traits are substantially heritable. Twin studies estimated the heritability of BPD to be 62% (Wray & Gottesman, 2012); they estimated around 60% for metabolic traits and around 40% for cardiovascular traits (Polderman et al., 2015). SNP-based heritability for BPD was estimated between 11% and 25% (Stahl et al., 2019) and estimates for cardiometabolic diseases ranged from 38% for stroke (Bevan et al., 2012), 40% for coronary artery disease (Nikpay et al., 2015), to 63% for type 2 diabetes [DIAbetes Genetics Replication Meta-analysis (DIAGRAM) Consortium et al., 2014). Recent genome-wide association studies (GWAS) identified 30 distinct loci associated with BPD (Stahl et al., 2019) and many loci associated with cardiometabolic traits [e.g. 304 loci for coronary artery disease (Nelson et al., 2017), 32 for stroke (Malik et al., 2018), 343 for type 2 diabetes (Mahajan et al., 2018)].

BPD and cardiometabolic traits are substantially heritable. Twin studies estimated the heritability of BPD to be 62% (Wray & Gottesman, 2012); they estimated around 60% for metabolic traits and around 40% for cardiovascular traits (Polderman et al., 2015). SNP-based heritability for BPD was estimated between 11% and 25% (Stahl et al., 2019) and estimates for cardiometabolic diseases ranged from 38% for stroke (Bevan et al., 2012), 40% for coronary artery disease (Nikpay et al., 2015), to 63% for type 2 diabetes [DIAbetes Genetics Replication Meta-analysis (DIAGRAM) Consortium et al., 2014). Recent genome-wide association studies (GWAS) identified 30 distinct loci associated with BPD (Stahl et al., 2019) and many loci associated with cardiometabolic traits [e.g. 304 loci for coronary artery disease (Nelson et al., 2017), 32 for stroke (Malik et al., 2018), 343 for type 2 diabetes (Mahajan et al., 2018)].

Despite the polygenicity of BPD and cardiometabolic traits, only a few population-based studies investigated their shared genetic aetiology. They revealed either no associations or suggested negative associations based on approaches analysing summary statistics. For example, using linkage disequilibrium score regression (LDSC), Stahl et al. (2019) found no genetic correlations between BPD and cardiometabolic traits, [body mass index (BMI), waist-to-hip ratio, type 2 diabetes, coronary artery disease], or lipid traits (cholesterol and triglycerides); nor did Hübel et al. (2019) between BPD and measures of body composition, including BMI. To date, the only study that systematically examined shared genetic aetiology between BPD and cardiometabolic traits found a reduced risk of BPD associated with *higher* polygenic scores for cardiometabolic traits (So, Chau, Ao, Mo, & Sham, 2019). Here, polygenic scores were calculated purely based on summary statistics and no individual-level data and therefore lacked some specificity. Associations were assessed using summary statistics for BMI, total cholesterol, triglycerides, waist-to-hip ratio and several other traits. LDSC did not reveal significant genetic correlations between the traits.

In summary, evidence for shared genetic aetiology between BPD and cardiometabolic traits in population-based samples is scarce. Their relationships, as well as their influencing factors, are poorly understood; no comprehensive, *individual-level* phenotypic and genetic investigation has been conducted.

Here, we investigate the relationship between BPD and cardiometabolic traits on a phenotypic and genetic level in the European UK Biobank (UKB) sample. First, we hypothesise that there is an increased risk of cardiometabolic traits in BPD. We assess phenotypic associations between BPD and three categories of cardiometabolic traits which were selected because they have been shown to be associated with BPD in the existing literature (Correll et al., 2017*a*; McIntyre et al., 2007; Prieto et al., 2014; Sinha et al., 2018; Vancampfort et al., 2015, 2016): anthropometric risk factors, biomarkers and cardiometabolic diseases. Second, we hypothesise that these polygenic traits share a genetic basis with BPD. We use two approaches; individual-level polygenic risk score (PRS) analyses and summary-level genetic correlations, to investigate shared genetic aetiology between BPD and the cardiometabolic traits.

## Methods

### Sample

The UKB is a prospective health resource including more than 500 000 people from across the UK (https://www.ukbiobank.ac.uk/) (Sudlow et al., 2015). More information about the cohort can be found in Supplementary Material 1.

### Phenotypic measures

#### Bipolar disorder (BPD)

BPD status was determined based on self-report questionnaires, a nurse interview and hospital episode statistics. BPD cases matched diagnostic ICD-10 codes for BPD (items 41202 and 41204; Supplementary Material 6), had a self-report diagnosis of mania, BPD or manic depression at nurse interview (items 20002 and 20544), fulfilled BPD criteria according to the baseline questionnaire of mood disorders (Smith et al., 2013), or fulfilled lifetime depression and mania criteria based on the Mental Health Questionnaire (Davis et al., 2019). Super-healthy controls, as defined by Glanville et al. (2020) (*MHQ controls*), did not meet any of these BPD criteria or any other mental health condition (ICD-9, ICD-10, self-report items 20002 and 20544, MHQ, Smith definition). Controls excluded participants with self-reported antipsychotic, antidepressant and mood stabiliser usage (item 20003).

Antidepressant, antipsychotic and mood stabiliser medication intake variables were based on self-reports (item 20003); mood stabiliser codes are in Supplementary Material 8 and all other medication codes in Glanville et al. (2020).

#### Blood pressure

Systolic and diastolic blood pressure was corrected for antihypertensive medication based on the recommendations by Tobin, Sheehan, Scurrah, and Burton (2005). If medication intake was reported (items 6177 and 6153), we added 15 mmHg to systolic blood pressure and 10 mmHg to diastolic blood pressure readings. Hypertension was determined based on high systolic and diastolic blood pressure measures (systolic ⩾140 mmHg and diastolic ⩾90 mmHg).

#### Body mass index

Two measures of BMI were available, one as weight/height (kg/m^2^) and the other measured using electrical impedance. Where both measures were available, we calculated the average of the measures and excluded participants who differed between the two measures by over 4.56 standard deviations (*n* = 1164) (Yaghootkar et al., 2016). If only one measure was available (*n* = 2923), this value was used.

#### Waist-to-hip ratio

Waist-to-hip ratio was calculated as the ratio between waist and hip circumference (cm/cm). The ratio was residualised for BMI to represent abdominal body mass distribution independent of overall body mass.

#### Biomarkers

Five blood biomarkers were analysed: lipid levels [total cholesterol, low-density lipoprotein cholesterol (LDL cholesterol), high-density lipoprotein cholesterol (HDL cholesterol) and triglycerides] and glycated haemoglobin (HbA1c). Quality control was performed by UKB using standardised laboratory procedures (Sinnott-Armstrong et al., 2019) (http://biobank.ndph.ox.ac.uk/showcase/showcase/docs/biomarker_issues.pdf). We corrected LDL and total cholesterol levels for individuals taking any cholesterol-lowering medication (Supplementary Material 7). LDL cholesterol levels for individuals taking medication were divided by 0.7, while total cholesterol levels were divided by 0.8 (Khera et al., 2016).

#### Type 2 diabetes

Type 2 diabetes cases were identified based on hospital episode statistics (ICD-9 and ICD-10), the national death register and self-reported questionnaires. Cases had self-reported type 2 or generic diabetes as established in the nurse interview and the touchscreen questionnaire. However, participants were only classified as cases when they reported in the questionnaire that (1) they had not been treated with insulin in the first year after diagnosis, (2) and had been diagnosed after the age of 35 years (Tyrrell, Yaghootkar, Freathy, Hattersley, & Frayling, 2013). Type 2 diabetes controls did not fulfil these type 2 diabetes criteria and did not have any other types of diabetes (inclusion and exclusion codes in Supplementary Material 9 and 10).

#### Coronary artery disease

Participants registered in the hospital in-patient data or the death register to have had ischemic heart diseases, or participants who had coronary revascularisation operations were classified as coronary artery disease cases (ICD-9 and 10 diagnoses and operation codes in Supplementary Material 11 and 12). Participants self-reporting those conditions in the nurse interview or touchscreen questionnaire were also considered coronary artery disease cases. Coronary artery disease controls did not fulfil coronary artery disease criteria.

#### Stroke

Stroke status was determined using stroke diagnoses in the hospital in-patient data and the death register. Additionally, participants self-reporting to have had a stroke in the nurse interview or in the touchscreen questionnaire were included as stroke cases (Schnier, Bush, Nolan, Sudlow, & on behalf of UK Biobank Outcome Adjudication Group, 2017). Stroke controls had no diagnosed or self-reported stroke.

Binary traits (BPD, type 2 diabetes, coronary artery disease, stroke) represent lifetime diagnoses (maximum three time points), and continuous variables were based on the first visit at the assessment centre. For a summary of items and codes, refer to Supplementary Material 5. Information on genotype quality control is in Supplementary Material 2.

### Statistical analyses

#### Phenotypic associations

To assess phenotypic relationships between BPD (*n*_cases_ = 4186; *n*_controls_ = 57 322) and cardiometabolic traits, we performed logistic regressions in R v3.6.0 (R Core Team, 2013). Continuous traits were standardised prior to analysis: they were total cholesterol, LDL and HDL cholesterol, triglycerides, HbA1c, diastolic and systolic blood pressure, BMI and waist-to-hip ratio, unadjusted and adjusted for BMI. All models were adjusted for assessment centre. The models regressing BPD onto biomarker variables were also adjusted for fasting time (models in Supplementary Material 14). We repeated this analysis for males (*n*_cases_ = 1926; *n*_controls_ = 30 039) and females (*n*_cases_ = 2260; *n*_controls_ = 27 283) separately to identify sex-specific associations. Phenotypic sensitivity analyses are described in Supplementary Material 3.

#### Polygenic risk score (PRS) regressions

We created PRSs representing genetic propensity to cardiometabolic traits by aggregating by-variant effects across the autosomes, weighted by effect sizes from GWAS summary statistics. PRSs were created for total cholesterol, LDL and HDL cholesterol, triglycerides (Willer et al., 2013), HbA1c (Wheeler et al., 2017), diastolic and systolic blood pressure (Evangelou et al., 2018), BMI (Locke et al., 2015), waist-to-hip ratio, unadjusted and adjusted for BMI (Shungin et al., 2015), coronary artery disease (Nikpay et al., 2015), stroke (Malik et al., 2018) and type 2 diabetes (Scott et al., 2017) (availability information in Supplementary Material 13). Every PRS was created at 11 *p-*value thresholds (PRS_PT_ = 5 × 10^−8^, 1 × 10^−5^, 0.001, 0.01, 0.05, 0.1, 0.2, 0.3, 0.4, 0.5, 1). PRSicev2 (https://github.com/choishingwan/PRSice) (Choi, Heng Mak, & O'Reilly, 2018; Euesden, Lewis, & O'Reilly, 2015) was used for clumping within windows of 250 kilobases (*r*^2^ < 0.25) and to create the PRSs. PRSs were standardised.

To test for shared genetic aetiology in individuals of European ancestries between BPD (*n*_cases_ = 4186; *n*_controls_ = 57 322) and cardiometabolic traits, we calculated logistic regressions with BPD as the dependent variable, and the standardised cardiometabolic PRSs at 11 *p*-value thresholds. The models were adjusted for assessment centre, genotyping batch and six genetic principal components to control for population stratification. This analysis was repeated for males (*n*_cases_ = 1926; *n*_controls_ = 30 039) and females (*n*_cases_ = 2260; *n*_controls_ = 27 283) separately to identify sex-specific associations.

Explained variances in the phenotypic and PRS analyses were calculated using Nagelkerke's pseudo-*R*^2^ and transformed to liability scale using a population prevalence of 8% (Cerimele, Chwastiak, Dodson, & Katon, 2014). Sensitivity analyses for PRS associations are described in Supplementary Material 4.

#### Genetic correlations

Genetic correlations were calculated between BPD and cardiometabolic traits using LDSC with the default HapMap LD reference (https://github.com/bulik/ldsc) (Bulik-Sullivan et al., 2015). We used the most recent GWAS summary statistics for BPD (Stahl et al., 2019), total cholesterol, LDL cholesterol, HDL cholesterol, triglycerides (Willer et al., 2013), HbA1c (Wheeler et al., 2017), diastolic and systolic blood pressure (Evangelou et al., 2018), BMI (Yengo et al., 2018) and waist-to-hip ratio, unadjusted and adjusted for BMI (Shungin et al., 2015), coronary artery disease (Nikpay et al., 2015), stroke (Malik et al., 2018) and type 2 diabetes (Scott et al., 2017) (availability of summary statistics in Supplementary Material 13). Genetic correlations calculated with LDSC are robust to sample overlap.

#### Correction for multiple testing

Correction for multiple testing was done for every separate analysis described above with a false discovery rate of 5% (Benjamini & Hochberg, 1995). *p*-values ⩽5.8 × 10^−4^ were considered significant in the primary phenotypic analyses, and *p*-values ⩽0.0065 in the primary PRS analyses.

## Results

The sample consisted of 61 508 participants (48% females), with a mean age of 57 years (s.d. = 7.69). The sample included 4186 BPD cases and 57 322 unaffected controls (mean age BPD group = 55; mean age controls = 57). Descriptive statistics of cardiometabolic traits by BPD case–control status are shown in Table 1 (descriptive statistics stratified by sex in online Supplementary Table S1, phenotypic relationships among cardiometabolic traits in online Supplementary Table S9).

**Table 1.** Descriptive statistics of the sample and phenotypic associations between BPD and cardiometabolic traits

Cardiometabolic traits	All participants (*N* = 61 508)	BPD cases (*n* = 4186)	Controls (*n* = 57 322)	Odds ratio	95% CI	*p* value	*R*^2^ (%)^a^
*Continuous traits: mean* (s.d.)							
Total cholesterol	5.73 (1.11)	5.67 (1.17)	5.74 (1.1)	**0.94**	**0.91–0.97**	**1.08 × 10^−4^**	**0.04**
LDL cholesterol	3.74 (0.83)	3.76 (0.9)	3.74 (0.82)	1.01	0.98–1.04	0.552	−0.03
HDL cholesterol	1.48 (0.38)	1.41 (0.39)	1.48 (0.38)	**0.81**	**0.78–0.84**	**1.73 × 10^−29^**	**0.67**
Triglycerides	1.73 (1.04)	1.99 (1.23)	1.71 (1.03)	**1.24**	**1.21–1.27**	**1.10 × 10^−56^**	**0.97**
HbA1c	35.27 (5.45)	35.83 (7.05)	35.23 (5.31)	**1.09**	**1.06–1.12**	**2.40 × 10^−10^**	**0.08**
Systolic blood pressure^b^	140.15 (20.04)	136.81 (19.77)	140.4 (20.04)	**0.82**	**0.80–0.85**	**9.47 × 10^−31^**	**0.59**
Diastolic blood pressure^b^	83.69 (11.08)	83.6 (11.3)	83.7 (11.06)	0.99	0.96–1.02	0.492	0.01
Body mass index	26.56 (4.25)	28.11 (5.38)	26.45 (4.14)	**1.39**	**1.35–1.43**	**1.70 × 10^−121^**	**2.18**
Waist-to-hip ratio	0.87 (0.09)	0.88 (0.09)	0.86 (0.09)	**1.22**	**1.18–1.26**	**1.14 × 10^−34^**	**0.66**
Waist-to-hip ratio adjusted for BMI	0 (0.08)	0.01 (0.08)	0 (0.08)	**1.06**	**1.02–1.09**	**5.79 × 10^−4^**	**0.06**
*Binary traits: affected cases, n* (%)							
Hypertension	14 821 (24.1)	963 (23.01)	13 858 (24.18)	0.93	0.87–1.01	0.069	0.02
Coronary artery disease	3188 (5.18)	373 (8.91)	2815 (4.91)	**1.89**	**1.69–2.12**	**3.75 × 10^−28^**	**0.45**
Stroke	854 (1.39)	127 (3.03)	727 (1.27)	**2.44**	**2.01–2.96**	**9.74 × 10^−20^**	**0.29**
Type 2 diabetes	1572 (2.56)	227 (5.42)	1345 (2.35)	**2.4**	**2.07–2.78**	**6.69 × 10^−32^**	**0.61**

Odds ratios indicate increased or decreased chances of having BPD, associated with an increase of one standard deviation in the cardiometabolic trait. Results with a false discovery rate of 5% at *p* ≤ 5.8 × 10^−4^ are in bold.

a *R*^2^ (%) indicates the percentage of explained variance in BPD by the cardiometabolic trait, calculated using Nagelkerke's pseudo-*R*^2^ and transformed to liability scale (population prevalence 8%). The negative *R*^2^ for LDL cholesterol was possible to obtain because we calculated *R*^2^ as the difference between full explained variance by a model including the cardiometabolic trait and all covariates (assessment centre, and for biomarkers also fasting time) and the explained variance by a model including covariates only.

b Diastolic and systolic blood pressure were adjusted for blood pressure medication (see 'Methods' section).

### Phenotypic associations

**Fig. 1.**
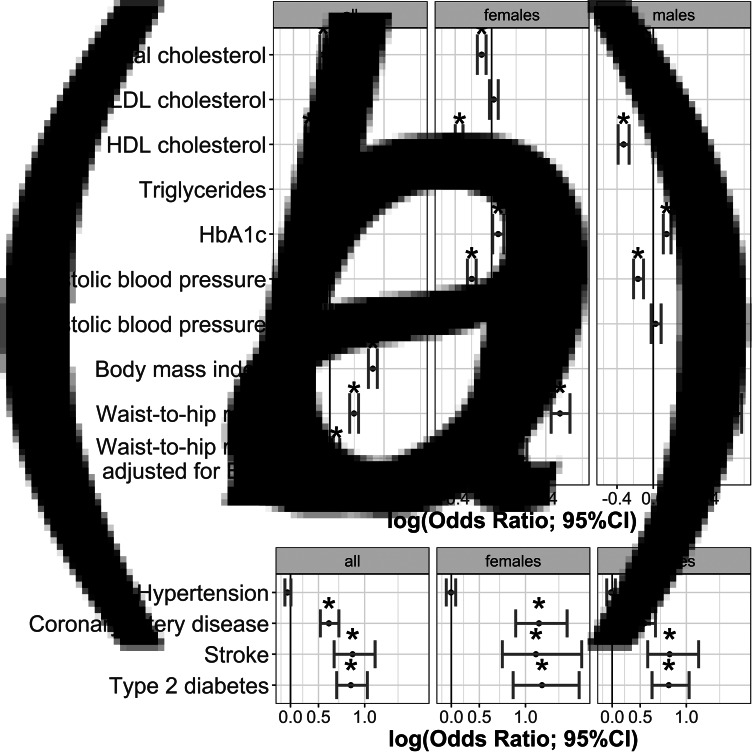
Odds ratios indicating the phenotypic associations between BPD and cardiometabolic traits. Significant associations at *p* ≤ 5.8 × 10^−4^ are marked with *. Panel (*a*) indicates odds ratios for the continuous traits and (*b*) odds ratios for the binary traits.

The phenotypic relationship between BPD and cardiometabolic traits was assessed using logistic regressions (Table 1). Meeting multiple testing correction of *p* ≤ 5.8 × 10^−4^ (FDR 5%), we observed positive associations between BPD and the cardiometabolic traits triglycerides, HbA1c, BMI, waist-to-hip ratio, waist-to-hip ratio adjusted for BMI, stroke, type 2 diabetes and coronary artery disease. Total cholesterol, HDL cholesterol and systolic blood pressure were negatively associated with BPD (Table 1, Fig. 1). The same analyses conducted separately for males and females showed consistent results with partly differing sex-specific effect sizes (e.g. CAD in males: OR = 1.74; females: OR = 3.03; full results in online Supplementary Tables S2 and S3).

#### Phenotypic sensitivity analysis

(1) To analyse whether phenotypic associations with blood pressure traits were biased by the blood pressure medication adjustment, phenotypic associations were re-calculated excluding participants on blood pressure medication. Systolic blood pressure remained negatively associated (OR = 0.75, 95% CI 0.72–0.79). Diastolic blood pressure (OR = 0.97, 95% CI 0.93–1.00) and hypertension (OR = 0.88, 95% CI 0.80–0.98) showed no effect on BPD, indicating that this association was independent of medication intake. (2) When limiting BPD cases to hospital in-patients only (*n*_cases_ = 920), odds ratios indicated larger effects (e.g. stroke OR increased from 2.44 to 4.94), with larger explained variances (e.g. BMI *R*^2^ increased from 2.81% to 3.70%; online Supplementary Table S7). (3) Adding antipsychotic, antidepressant and mood stabiliser medication as covariates showed consistent directions of effects and largely the same significant results between cardiometabolic traits and BPD as found in the primary analysis (online Supplementary Tables S4, S5, S6). However, odds ratios decreased and explained variances reduced drastically (e.g. BMI from *R*^2^ = 2.18 in primary analysis to 0.83 when controlling for antidepressants). Waist-to-hip ratio adjusted for BMI became non-significant when controlling for either medication, and had the largest decrease in odds ratios from 1.06 (95% CI 1.02–1.09) in primary analysis to 1.00 (95% CI 0.96–1.03) when controlled for mood stabilisers. (4) Residualising CMTs for age and re-calculating their association with BPD yielded similar results to the primary associations; they can be found in online Supplementary Table S8.

### Polygenic risk score analysis

**Fig. 2.**
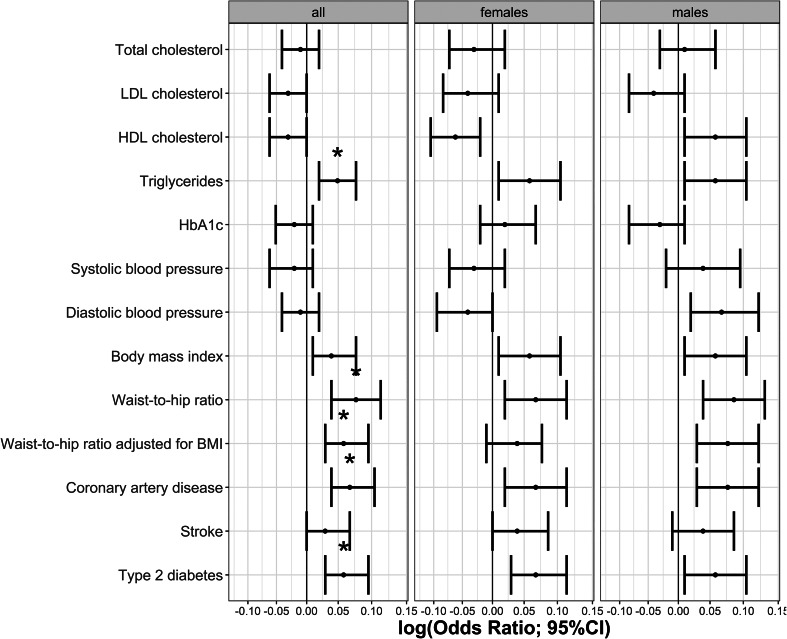
Association between BPD and the most predictive PRSs for each cardiometabolic trait. Significant associations at *p* ≤ 0.0065 are marked with *.

Logistic regressions were performed to test the association between BPD and PRSs at 11 *p*-value thresholds for each cardiometabolic trait. Results for the *most predictive* thresholds in each cardiometabolic trait are indicated in Table 2 and Fig. 2 (we observed at least four out of 11 significantly associated PRS thresholds for every significant trait). At a multiple testing correction of *p* ≤ 0.0065 (FDR 5%), BPD was positively associated with PRSs for triglycerides, waist-to-hip ratio, waist-to-hip ratio adjusted for BMI, coronary artery disease and type 2 diabetes. The highest contribution to explaining variance in BPD was from the waist-to-hip ratio PRS (PRS_PT_ = 0.5) accounting for 0.13% of the variance (full results in online Supplementary Table S10). PRS analyses repeated for males and females separately did not survive correction for multiple testing; however, directions of effects were largely consistent with the primary analysis (online Supplementary Tables S11 and S12).

**Table 2.** Associations between BPD and PRS for each cardiometabolic traits at the most predictive threshold

Cardiometabolic trait	PRS_PT_^a^	Odds ratio	95% CI	*p* value	*R*^2^ (%)^b^
*Biomarkers*					
Total cholesterol	0.01	0.99	0.96–1.02	0.5100	0.00
LDL cholesterol	0.01	0.97	0.94–1	0.0497	0.02
HDL cholesterol	0.5	0.97	0.94–1	0.0724	0.02
**Triglycerides**	0.4	**1.05**	**1.02–1.08**	**0.0028**	**0.06**
HbA1c	0.001	0.98	0.95–1.01	0.2720	0.01
*Anthropometric traits*					
Systolic blood pressure	0.01	0.98	0.94–1.01	0.1520	0.01
Diastolic blood pressure	0.001	0.99	0.96–1.02	0.4810	0.00
Body mass index	0.4	1.04	1.01–1.08	0.0142	0.04
**Waist-to-hip ratio**	0.5	**1.08**	**1.04–1.12**	**4.50 × 10^−6^**	**0.13**
**Waist-to-hip ratio adjusted for BMI**	0.3	**1.06**	**1.03–1.1**	**0.0003**	**0.08**
*Cardiometabolic diseases*					
**Coronary artery disease**	1	**1.07**	**1.04–1.11**	**2.03 × 10^−5^**	**0.11**
Stroke	0.1	1.03	1–1.07	0.0464	0.02
**Type 2 diabetes**	0.4	**1.06**	**1.03–1.1**	**0.0003**	**0.08**

Results with a false discovery rate of 5% at *p* ≤ 0.0065 are printed in bold. The table only includes the most predictive PRS_PT_ for each trait. Associated traits showed significant associations at a minimum of four PRS thresholds; full results in online Supplementary Table 10. Odds ratios indicate changes in chances of having BPD associated with an increase of one standard deviation in the PRS.

a PRS_PT_ indicates the *p*-value threshold used to create the PRSs.

b *R*^2^ (%) indicates the percentage of explained variance in BPD and was calculated using Nagelkerke's pseudo *R*^2^, transformed to liability scale (population prevalence 8%). The indicated *R*^2^ is the difference between full explained variance by a model including the cardiometabolic trait and all covariates (assessment centre, batch and six genetic PCs) and the explained variance by a model including covariates only.

#### Sensitivity analyses for PRS associations

(1) To assess whether significant PRS associations were driven by phenotypic associations between BPD and cardiometabolic traits, we investigated the attenuation of explained variance when controlling for the base cardiometabolic phenotype. For binary traits, we re-calculated associations for significant PRSs with BPD by excluding cases with the corresponding cardiometabolic disease (i.e. T2D analyses: excluding 1572 T2D cases; CAD analyses: excluding 3188 CAD cases). For coronary artery disease PRSs, explained variance was hardly attenuated. For example, the variance explained with PRS_PT_ 0.4 declined from *R*^2^ = 0.081% in the main analysis to *R*^2^ = 0.076% in the sensitivity analysis (relative attenuation ⩽5.90%). When excluding type 2 diabetes cases, type 2 diabetes PRS associations had a substantial attenuation in explained variance. For example, for PRS_PT_ 0.4 it declined from *R*^2^ = 0.080 in the main analysis to *R*^2^ = 0.059% in the sensitivity analysis (relative attenuation 15–26%; online Supplementary Table S16). For continuous traits, we repeated the PRS analyses adding the base cardiometabolic trait as a covariate; that is, triglycerides, and both waist-to-hip ratios, respectively. In those three cases, explained variance drastically declined. The explained variance by triglycerides PRS (PRS_PT_ 1) dropped from *R*^2^ = 0.049% in the primary analysis to *R*^2^ = 0.00008% when adding triglycerides as a covariate (relative attenuation in *R*^2^ across thresholds between 96% and 100%). Explained variance by waist-to-hip ratio PRS (PRS_PT_ 0.1) declined from *R*^2^ = 0.055% in primary analysis to *R*^2^ = 0.021% (relative attenuation: 53–62%), and for waist-to-hip ratio adjusted for BMI PRS (PRS_PT_ 0.2), a relatively smaller decrease from *R*^2^ = 0.055% to *R*^2^ = 0.046% (relative attenuation in *R*^2^ = 14–17%; online Supplementary Table S17). (2) To test whether the strength of the phenotype influenced the associations, we restricted to BPD cases based on hospital in-patient ICD10 diagnoses (*n*_cases_ = 920). None of these associations survived correction for multiple testing (online Supplementary Table S13). (3) Predictive abilities of PRSs are displayed in online Supplementary Tables S14 and S15. (4) There were no significant associations when predicting cardiometabolic traits using BPD PRS (online Supplementary Tables S18 and S19).

### Genetic correlations

**Fig. 3.**
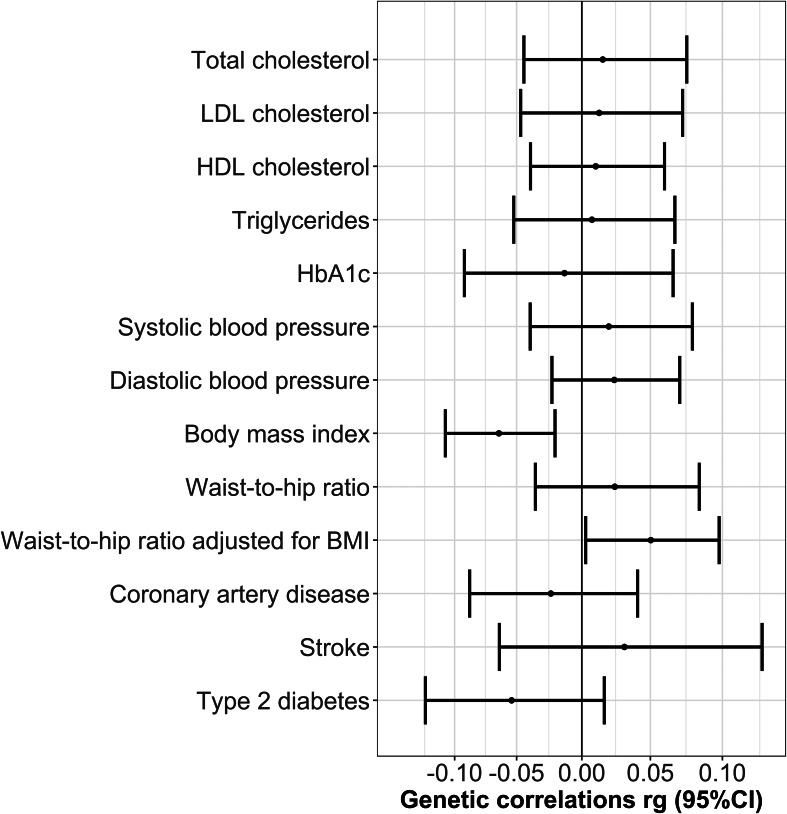
Genetic correlations between BPD and cardiometabolic traits.

Genetic correlations between BPD and cardiometabolic traits were low and not significant after correcting for multiple testing (Table 3, Fig. 3). BMI (*r_g_* = −0.064, *p* = 0.004) and waist-to-hip ratio adjusted for BMI (*r_g_* = 0.050, *p* = 0.038) were nominally correlated but did not survive correction for multiple testing.

**Table 3.** Genetic correlations between BPD and 13 cardiometabolic traits calculated using LDSC regression

Cardiometabolic trait	*h*2	*h*2 *se*	*r_g_*	95% CI	*p*-value
*Biomarkers*					
Total cholesterol	0.2472	0.0384	0.016	−0.044 to 0.076	0.611
Triglycerides	0.2857	0.0620	0.008	−0.052 to 0.067	0.805
HDL cholesterol	0.2474	0.0388	0.010	−0.039 to 0.060	0.681
LDL cholesterol	0.2209	0.0466	0.013	−0.047 to 0.073	0.673
HbA1c	0.0627	0.0088	−0.013	−0.092 to 0.066	0.747
*Anthropometric traits*					
Body mass index	0.2163	0.0070	−0.064	−0.108 to −0.020	0.004
Waist-to-hip ratio	0.1047	0.0067	0.024	−0.035 to 0.084	0.423
Waist-to-hip ratio (adjusted for BMI)	0.1207	0.0081	0.050	0.003–0.098	0.038
Systolic blood pressure	0.1159	0.0068	0.020	−0.039 to 0.079	0.509
Diastolic blood pressure	0.1413	0.0065	0.024	−0.023 to 0.071	0.314
*Cardiometabolic diseases*					
Stroke	0.0121	0.0015	0.031	0.064–0.126	0.517
Coronary artery disease	0.0681	0.0053	−0.023	−0.088 to 0.041	0.475
Type 2 diabetes	0.0792	0.0061	−0.054	−0.124 to 0.017	0.134

SNP-heritability for BPD estimated using LDSC = 0.342 (s.e. = 0.019).

## Discussion

This study investigated the relationship between BPD and cardiometabolic traits in UKB on a phenotypic and genetic level, using PRS analyses and genetic correlations.

### Phenotypic associations

The phenotypic results revealed significant, but weak associations with several cardiometabolic traits, namely low total cholesterol, high triglycerides, low HDL cholesterol, low systolic blood pressure, high BMI, high waist-to-hip ratio, as well as stroke, type 2 diabetes and coronary artery disease status. These findings were largely consistent with our hypothesis that BPD status is associated with increased cardiometabolic risk.

Associations with biomarkers were consistent with meta-analytic findings for triglycerides, HDL cholesterol, HbA1c (Vancampfort et al., 2013, 2015) and BMI (Correll et al., 2017*b*). We are unaware of previous studies reporting increased relative risk for BPD conveyed by waist-to-hip ratio unadjusted and adjusted for BMI. These findings indicate that, on average, there was increased body mass in BPD cases, which was found for body fat distribution and abdominal obesity independent of body fat. Coronary artery disease, stroke and type 2 diabetes were associated with at least twofold increased odds of having BPD, which converges with epidemiological meta-analyses (Correll et al., 2017*b*; Vancampfort et al., 2016).

In contrast, we observed surprising directions of effects in associations with BPD for total cholesterol and blood pressure. Total cholesterol was lower in BPD cases than in controls. This contradicts our hypothesis that cardiometabolic risk is increased in BPD. The literature assessing total cholesterol in BPD is scarce; one study showed no effect of peripheral total cholesterol on BPD (Garcia-Portilla et al., 2009).

It was unexpected, based on the unhealthy effects of total cholesterol demonstrated in other samples, that we found unusual relationships between total cholesterol and other cardiometabolic traits. For example, it was lowered in participants with coronary artery disease, stroke and type 2 diabetes. This could be indexing a survivor bias, meaning that participants within the UKB demonstrate health-risk factors, such as high cholesterol, but are otherwise unusually healthy, introducing spurious relationships between variables (Schooling, 2019). As the association with LDL and HDL cholesterol showed directions of effects as expected from previous literature, future studies could consider those potentially more robust variables instead of a composite total cholesterol measure in the context of BPD.

The survivor bias is also reflected in significant age differences [*t*(61 506) = 17.92, *p* < 0.0001) between BPD cases (mean = 54.58, s.d. = 7.93) and controls (mean = 56.78, s.d. = 7.65)]. Controls are likely older because participants must remain healthy until the last measured time point to classify as controls. However, associations between cardiometabolic traits and BPD in this study remained robust when controlled for age. Future studies should further assess these associations and potential age-related influences using longitudinal BPD definitions.

We observed a negative association between BPD and systolic blood pressure, whereas diastolic blood pressure and hypertension were not associated. This was unexpected because elevated systolic and diastolic measures were both linked with negative health outcomes, including cardiovascular events (Cooper-DeHoff et al., 2010). Hypertension was previously shown to be increased in BPD cases (Ayerbe et al., 2018); and further investigation in other samples is needed to clarify whether this effect is real.

When calculating associations with BPD for males and females separately, we found largely consistent, but partly differing sex-specific effect sizes. Future studies should split their analyses by sex for more accurate estimates and discern whether differences rely on noise or sex-specific mechanisms, particularly for coronary artery disease.

Sensitivity analyses restricted to more severe BPD cases (*n*_cases_ = 920) confirmed associations found in primary analyses and explained more variance. This strengthens the evidence that the significant phenotypic associations are not artefactual, because these 920 cases are classified with high confidence having been admitted to hospital for BPD. It is possible that these stronger associations reflect that cardiometabolic traits themselves increase the likelihood for BPD participants to seek medical help and receive a BPD diagnosis.

Finally, we controlled the associations between BPD and cardiometabolic traits for antipsychotic, antidepressant and mood stabiliser usage. Directions of effects were consistent compared with the primary analyses, but odds ratios and explained variances were considerably lower suggesting that medications moderate the associations and that effects depend on medication intake. For example, the *R*^2^ of the BMI–BPD association attenuated from 2.18% to 0.83% when controlling for antidepressants; and the WHR–BPD association attenuated from 0.66% to 0.16% when controlling for mood stabilisers. However, it must be considered that medication self-reports only concerned 2-week windows and might not represent long-term intake. Adding them as covariates probably added some precision but might not have entirely ruled out influences by medication intake. Future studies with access to more detailed information about medication usage (for example, electronic health records) could further examine the effect of medication use on the association between BPD and cardiometabolic traits.

### Shared genetic aetiology

We discovered evidence for shared genetic aetiology in European ancestries based on PRS associations in primary analyses for five cardiometabolic traits: triglycerides, waist-to-hip ratio unadjusted and adjusted for BMI, coronary artery disease and type 2 diabetes. Associations re-calculated for males and females showed consistent directions of effects but did not survive correction for multiple testing; nor did they when cases were limited to more severe BPD cases (*n*_cases_ = 920). The number of cases was probably underpowered to capture significant PRS associations, underlining the importance of future large-scale studies.

The small, explained variances found in the primary PRS analyses could be due to the limited predictive power of the PRSs, which was demonstrated when using PRSs to predict their base cardiometabolic phenotype. The best prediction was for LDL cholesterol by LDL cholesterol PRS with up to 11% explained variance. The lowest prediction was by stroke PRS which explained 0.12% variance in the stroke phenotype, reflecting the small sample size used to calculate the stroke GWAS. Future studies will benefit from larger GWAS and better predictive power of PRSs.

None of the PRS associations were replicated by genetic correlations inferred through LDSC. As genetic correlations are calculated with GWAS summary statistics and PRS analyses rely on individual-level genotype data, the PRS analyses might have been more powerful (genetic correlations have larger standard errors; van Rheenen, Peyrot, Schork, Lee, & Wray, 2019). Therefore, PRS associations could have captured real shared genetic aetiology undetected by LDSC. This could explain the discrepancy with the findings by So et al. (2019), as their PRS analyses did not consider individual-level genotype data. It is equally possible that our results reflect discontinuity between the UKB phenotypes compared with PGC; and our findings might describe unrepresentative BPD relationships specific to UKB.

Furthermore, we investigated whether associations between cardiometabolic PRSs and BPD remained significant after controlling for the base phenotype, either by excluding participants with the cardiometabolic disease or by adding the cardiometabolic phenotype as a covariate in the model. We observed different degrees of attenuation in explained variances, including very little relative attenuation in the case of coronary artery disease (average attenuation 1.09%), and full relative attenuation for triglycerides (average attenuation 98.76%). This indicates that, when predicting BPD, adding a coronary artery disease PRS seems to explain some additional variance beyond the variance captured by the coronary artery disease phenotype. This indicates shared genetic aetiology with BPD above and beyond their base phenotypes in UKB, which was not the case for triglycerides, and at least partly applied to type 2 diabetes, waist-to-hip ratio unadjusted and adjusted for BMI. Consistent with phenotypic associations reported above, these differing patterns indicate distinct relationships with BPD for individual cardiometabolic traits, some of which probably share some genetic aetiology in UKB with BPD; mainly type 2 diabetes, coronary artery disease and waist-to-hip ratio. Future research is imperative to investigate to what extent those results are prone towards collider bias (Akimova, Breen, Brazel, & Mills, 2020).

BPD PRSs did not significantly predict any cardiometabolic phenotypes, which could be due to differences in statistical power between the BPD GWAS and the CMT GWAS. This underlines the need for future more powerful BPD GWAS that will facilitate to disentangle whether the genetic overlap with genetic underpinnings of CMTs relies on shared biological pathways, pleiotropy or whether CMTs confound BPD GWAS summary statistics.

### Limitations

There are additional limitations, beyond those highlighted throughout this discussion. The *possible bipolar* status definition was chosen for its high predictive validity (Davis et al., 2019). However, it outlines a non-clinical subpopulation with increased prevalence of BPD symptoms and caution must be taken when translating our findings. Because of a healthy-volunteer selection bias, particularly among participants in the Mental Health Questionnaire, it is unclear how generalisable findings are (Adams et al., 2018). Selection bias probably introduced spurious relationships for variables that influence participation in UKB (Munafò, Tilling, Taylor, Evans, & Davey Smith, 2017). Phenotypic and PRS associations explained only a fraction of variance in BPD (*R*^2^ phenotypes ≤2.18%; PRS ≤0.13%), and none implicated causal biological mechanisms.

## Conclusion

This large-scale, cross-sectional study constitutes a nuanced account of the comorbidity between BPD and various, extensively phenotyped, cardiometabolic traits, considering clinical and self-report data, physical measurements, and biological samples. BMI was most strongly associated with an increased risk of BPD. We found shared genetic aetiology for coronary artery disease, type 2 diabetes, and waist-to-hip ratio unadjusted and adjusted for BMI and triglycerides. These associations only persisted beyond the variance explained by cardiometabolic base phenotypes in the case of coronary artery disease. This was not replicated by genetic correlations, accentuating the importance of choosing appropriate statistical techniques when testing for shared genetic aetiology.

Our results underline that cardiometabolic comorbidity in BPD differs between cardiometabolic traits, where effect sizes depend on sex, medication intake and the phenotype strength. This should motivate future hypothesis-driven longitudinal research inside and outside UKB to consider specific cardiometabolic traits for associations with BPD, rather than an overarching global metabolic trait, when attempting to disentangle underlying biological mechanisms.

## Supplementary material

For supplementary material accompanying this paper visit https://doi.org/10.1017/S0033291721000945. click here to view supplementary material
