# Supplementary material for: Associations and limited shared genetic aetiology between bipolar disorder and cardiometabolic traits in the UK Biobank
Source: Psychol Med. 2021 Mar 26;52(16):4039–48. doi: 10.1017/S0033291721000945 (PMC9811277; doi:10.1017/S0033291721000945)
Supplement: Supplementary file 1 [file S0033291721000945sup.zip › S0033291721000945sup001.docx]

**Supplementary materials**

Contents

[*Supplementary Material 1.* Description of the UK Biobank cohort 2](#_Toc54968963)

[*Supplementary Material 2.* Genotype quality control 2](#_Toc54968964)

[*Supplementary Material 3.* Phenotypic sensitivity analyses 3](#_Toc54968965)

[*Supplementary Material 4.* Sensitivity analyses for PRS associations 4](#_Toc54968966)

[*Supplementary Material 5.* Table methods phenotype definition 5](#_Toc54968967)

[*Supplementary Material 6.* Bipolar disorder ICD-10 codes used in to define BPD cases 7](#_Toc54968968)

[*Supplementary Material 7*. UKB medication codes used to adjust biomarkers for medication intake 7](#_Toc54968969)

[*Supplementary Material 8.* UKB medication codes for mood stabilisers used to create super-healthy BPD controls (UKB item 20003) 8](#_Toc54968970)

[*Supplementary Material 9.* Type 2 diabetes ICD-9 and ICD-10 codes used to define cases in this study 9](#_Toc54968971)

[*Supplementary Material 10.* Other diabetes ICD-9 and ICD-10 codes used to exclude participants from cases and controls in this study 10](#_Toc54968972)

[*Supplementary Material 11.* Coronary artery disease ICD-9 and ICD-10 codes used in this study 12](#_Toc54968973)

[*Supplementary Material 12.* Operation codes used to define coronary artery disease 14](#_Toc54968974)

[*Supplementary Material 13.* Availability of GWAS summary statistics used to create polygenic risk scores and calculate genetic correlations. 16](#_Toc54968975)

[*Supplementary Material 14.* Statistical models used for statistical analysis 17](#_Toc54968976)

Supplementary Tables 1-15 are displayed in a separate Excel document.

# *Supplementary Material 1.* Description of the UK Biobank cohort

The UK Biobank (UKB) is a prospective cohort study including more than 500,000 people from across the United Kingdom (<https://www.ukbiobank.ac.uk/>) (Sudlow et al., 2015). It holds extensive health-related information from physical measurements, biological samples, and questionnaires, collected during visits to assessment centres and online follow-ups. Two questionnaires in the UKB assessed depressive and manic symptoms. A touchscreen questionnaire assessed “lifetime history of mood disorders” (Smith et al., 2013) and was completed by 172,751 participants at baseline; the second is the mental health online follow-up questionnaire (MHQ), conducted by 157,366 participants (Davis et al., 2020). In addition, hospital in-patient, national death register, and genome-wide genotyping data was available for all participants. The UKB study received ethical approval by the Research Ethics Committee. All participants signed informed consent.

# *Supplementary Material 2.* Genotype quality control

UKB genetic data was genotyped on the UK BiLEVE and the UK Biobank Axiom array and underwent centralised quality control resulting in samples with 805,426 genotype markers for 488,377 available participants (Bycroft et al., 2017). Genotype data used in this study excluded rare variants (minor allele frequency < 0.01). Further quality control has been described elsewhere (Coleman et al., 2019). Briefly, participants who withdrew consent, had uncommon levels of heterozygosity or high missingness (> 0.05) were excluded. Participants with low call rates for genotyped SNPs (< 98%) were removed, and those who were related to another participant in the dataset (KING statistic of r < 0.044) (Manichaikul et al., 2010). Relatives were removed based on a greedy algorithm (eg. by excluding the child in a mother-father-child trio). Participants with mismatches between phenotypically and genetically indicated sex were removed (X-chromosome homozygosity (F_X_) > 0.5 for phenotypic females, F_X_ < 0.9 for phenotypic males). Only participants of European ancestries were analysed, which was determined based on 4-means clustering on the first two genetic principal components (Warren et al., 2017). Six genetic principal components were identified to adjust for population stratification and technical artefacts in downstream analyses (Abraham, Qiu, & Inouye, 2017).

# *Supplementary Material 3.* Phenotypic sensitivity analyses

The following sensitivity analyses have been conducted to test for influences on phenotypic associations between BPD and various cardiometabolic traits found in the primary analyses described in the main manuscript. (1) To test whether adjustment for blood pressure medication influenced the associations, we recalculated them excluding individuals on blood pressure medication (*n* = 10,194). (2) We re-calculated the phenotypic associations only including BPD cases based on hospital in-patient data, thereby probably reflecting more severe cases (*n_cases_ = 920; n_controls_ = 63,162*). (3) We re-evaluated the associations between cardiometabolic traits and BPD in the full sample (*N* = 61,508) by adding self-reported antidepressant, antipsychotic and mood stabiliser usage separately as covariates. There was no indication of strong multicollinearity between BPD and medication variables; their variance inflation index was below 2 (VIF’s: mood stabilisers = 1.25, antipsychotics = 1.14, antidepressants = 1.40). (4) We residualised all cardiometabolic traits for age and calculated the association of those residualised variables with BPD. (5) We calculated associations among cardiometabolic traits: Pearson’s correlations for relationships between continuous traits, and logistic regressions to quantify odds ratios when at least one trait was binary.

# *Supplementary Material 4.* Sensitivity analyses for PRS associations

The following sensitivity analyses have been conducted to test for influences on associations between BPD and various PRSs for cardiometabolic traits found in the primary analyses described in the main manuscript. Explained variances were calculated using Nagelkerke’s pseudo-*R^2^* and transformed to liability scale using a population prevalence of 8% (Cerimele, Chwastiak, Dodson, & Katon, 2014). (1) To identify post-hoc whether significant PRS associations found in the primary analyses (*N* = 61,508) were driven by phenotypic associations between BPD and the cardiometabolic trait, modelling for binary traits was repeated excluding cases affected by the binary base cardiometabolic trait (association between BPD and type 2 diabetes: excluded 1,723 type 2 diabetes cases; association between BPD and coronary artery disease: excluded 3,505 coronary artery disease cases). Modelling for continuous traits was repeated by adding the base cardiometabolic phenotype as a covariate in the model. Relative attenuation in *R^2^* was calculated as the difference between original *R^2^* and the sensitivity *R^2^*, divided by the original *R^2^*. (2) PRS regressions were re-assessed employing a more conservative definition of BPD, including BPD cases based on hospital-in patient ICD10 codes only. A population prevalence of 1% was used for these analyses to transform Nagelkerke’s R2 to liability scale (Pini et al., 2005). (3) Predictive ability of cardiometabolic PRSs was assessed by calculating linear and logistic regressions predicting their base continuous and binary phenotypes, respectively. (4) Finally, we calculated BPD PRSs at eleven *p*-value thresholds (Stahl et al., 2019) and predicted cardiometabolic traits.

# *Supplementary Material 5.* Table methods phenotype definition

| **Trait** | **Self-report** (**UKB Item**: *codes*) | **Hospital in-patient data**  (**main & secondary** **UKB Item**: *codes*) | **Death register** (**main & secondary** **UKB Item**: *codes*) | **Operations OPS-4**  (**UKB Item**: *codes*) |
| --- | --- | --- | --- | --- |
| **Coronary artery disease** | - Non-cancer illness code, self-reported (item **20002**): heart attack/myocardial infarction (code *1075*) - Operation code (**20004**): coronary angioplasty (*1070*)*,* coronary artery bypass grafts (*1095*), triple heart bypass (*1523*) - Vascular/heart problems diagnosed by doctor (**6150**): heart attack (*1*), angina (*2*) | - Diagnoses - main ICD9 (**41203)** & diagnoses - secondary ICD9 (**41205)**: *410 - 414* - Diagnoses - main ICD10 (**41202**) & diagnoses - secondary ICD10 **(41204**): *I20 - I25* individual codes in Supplementary Material 7 | - Underlying (primary) cause of death: ICD10 (**40001)** & Contributory (secondary) causes of death: ICD10 (**40002**): *I20 - I25* | Operative procedures - main OPCS4 (**41200**): *K401-K469, K501, K751-K759* Supplementary Material 8 |
| **Stroke** | - Non-cancer illness code, self-reported (**20002**): stroke (*1081),* subarachnoid haemorrhage (*1086*), brain haemorrhage (*1491*), ischaemic stroke *(1583*) - Vascular/heart problems diagnosed by doctor (**6150**): stroke (*3*) | - Diagnoses - main ICD9 (**41203)** & diagnoses - secondary ICD9 (**41205):** *430 – 431, 434, 436* - Diagnoses - main ICD10 (**41202**) & diagnoses - secondary ICD10 **(41204**): I60 – I61, I63 – I64 individual codes in Schnier, Bush, Nolan, and Sudlow (2017) | - Underlying (primary) cause of death: ICD10 (**40001)** & Contributory (secondary) causes of death: ICD10 (**40002**): *I60 – I64* | NA |
| **Type 2 diabetes**  **(inclusion criteria)** | - Non-cancer illness code, self-reported (**20002**): type 2 diabetes (*1223*), diabetes (*1220*) - Diabetes diagnosed by doctor **(2443**), yes (*1*) | - Diagnoses - main ICD9 (**41203)** & diagnoses - secondary ICD9 (**41205):**  *25000, 25010, 25020, 25090* - Diagnoses - main ICD10 (**41202**) & diagnoses - secondary ICD10 **(41204**): *E11*  individual codes in Supplementary Material 5 | - Underlying (primary) cause of death: ICD10 (**40001)** & Contributory (secondary) causes of death: ICD10 (**40002**): *E11* | NA |
| **Type 2 diabetes**  **(exclusion criteria)** | - Non-cancer illness code, self-reported (**20002**): type 1 diabetes (*1222*), gestational diabetes (*1221*)*,* diabetes insipidus (*1521*) - Gestational diabetes only (**4041**): yes (*1*) - Started insulin within one year diagnosis of diabetes **(2986)**: yes (*1*) - Age diabetes diagnosed (**2976**): age >= 35 | - Diagnoses - main ICD9 (**41203)** & diagnoses - secondary ICD9 (**41205):**  *25011, 25021, 25091, 5881, 7751* - Diagnoses - main ICD10 (**41202**) & diagnoses - secondary ICD10 **(41204**): *E10, E12 - E14* individual codes in Supplementary Material 6 | - Underlying (primary) cause of death: ICD10 (**40001)** & Contributory (secondary) causes of death: ICD10 (**40002**): *E10, E12 - E14* | NA |

All items were considered at all three available time points.

# *Supplementary Material 6.* Bipolar disorder ICD-10 codes used in to define BPD cases

| **ICD-10 code** | | **UKBB code** | **Diagnosis** |
| --- | --- | --- | --- |
|  | *F31* | *I25* | *Bipolar affective disorder* |
|  | F31.0 | F310 | Bipolar affective disorder, current episode hypomanic |
|  | F31.1 | F311 | Bipolar affective disorder, current episode manic without psychotic symptoms |
|  | F31.2 | F312 | Bipolar affective disorder, current episode manic with psychotic symptoms |
|  | F31.3 | F313 | Bipolar affective disorder, current episode mild or moderate depression |
|  | F31.4 | F314 | Bipolar affective disorder, current episode severe depression without psychotic symptoms |
|  | F31.5 | F315 | Bipolar affective disorder, current episode severe depression with psychotic symptoms |
|  | F31.6 | F316 | Bipolar affective disorder, current episode mixed |
|  | F31.7 | F317 | Bipolar affective disorder, currently in remission |
|  | F31.8 | F318 | Other bipolar affective disorders |
|  | F31.9 | F319 | Bipolar affective disorder, unspecified |

ICD-9 diagnoses were not included as it does not define BPD as a distinct disorder. Diagnoses adapted directly from International Statistical Classification of Diseases (ICD-10; World Health Organization, 2004), as indicated on the UKB website.

# *Supplementary Material 7*. UKB medication codes used to adjust biomarkers for medication intake

| **UKB item** | Label | UKB code |
| --- | --- | --- |
| **6177** | Do you regularly take any of the following medications? | 1: Cholesterol lowering medication  2: Blood pressure medication  3: Insulin |
| **20003** | colestipol | 1140888590 |
|  | colestyramine | 1140909780 |
|  | colestyramine product | 1141180734 |
|  | ezetimibe | 1141192736 |
|  | ezetrol 10mg tablet | 1141192740 |
|  | supralib 160mg m/r tablet | 1141172214 |
|  | atorvastatin | 1141146234 |
|  | crestor 10mg tablet | 1141192414 |
|  | fluvastatin | 1140888594 |
|  | lescol 20mg capsule | 1140864592 |
|  | lipid lowering drug | 1140861922 |
|  | lipitor 10mg tablet | 1141146138 |
|  | lipostat 10mg tablet | 1140861970 |
|  | pravastatin | 1140888648 |
|  | rosuvastatin | 1141192410 |
|  | simvador 10mg tablet | 1141188146 |
|  | simvastatin | 1140861958 |
|  | zocor 10mg tablet | 1140881748 |
|  | ranzolont 10mg tablet | 1141195196 |
|  | zocor heart-pro 10mg tablet | 1141200040 |
|  | colestid 5g/sachet granules | 1140861848 |
|  | bezafibrate | 1140861924 |
|  | bezalip 200mg tablet | 1140861926 |
|  | questran 4g/sachet powder | 1140861936 |
|  | clofibrate | 1140861944 |
|  | fenofibrate | 1140861954 |
|  | ciprofibrate | 1140862026 |
|  | bezafibrate product | 1141157260 |
|  | lipantil micro 67mg capsule | 1141162544 |

# *Supplementary Material 8.* UKB medication codes for mood stabilisers used to create super-healthy BPD controls (UKB item 20003)

| **UKB name** | **Chemical name** | **UKB code** |
| --- | --- | --- |
| valproic acid | Valproic acid | 1140872214 |
| sodium valproate | Valproic acid | 1140872198 |
| convulex 150mg e/c capsule | Valproic acid | 1140872216 |
| depakote 250mg e/c tablet | Valproic acid | 1141172838 |
| epilim 100mg crushable tablet | Valproate | 1140872200 |
| epival cr 300mg m/r tablet | Valproate | 1141182592 |
| priadel 200mg m/r tablet | Lithium | 1140867504 |
| camcolit 250 tablet | Lithium | 1140867494 |
| liskonum 450mg m/r tablet | Lithium | 1140867498 |
| li-liquid 5.4mmol/5ml oral solution | Lithium | 1140867520 |
| li-liquid 509mg/5ml oral solution | Lithium | 1140917270 |
| lithium product | Lithium | 1140867490 |
| lamotrigine | lamotrigine | 1140872290 |
| lamictal 25mg tablet | lamotrigine | 1140872302 |
| carbamazepine | carbamazepine | 2038459704 |
| carbagen sr 200mg m/r tablet | carbamazepine | 1141171566 |
| tegretol 100mg tablet | carbamazepine | 1140872072 |
| carbamazepine | carbamazepine | 1140872064 |
| oxcarbazepine | oxcarbazepine | 1141175204 |
| trileptal 150 tablet | oxcarbazepine | 1141175212 |
| topiramate | topiramate | 1140923484 |
| topamax 25mg tablet | topiramate | 1140927692 |
| gabapentin | gabapentin | 1140872228 |
| neurontin 100mg capsule | gabapentin | 1140872236 |
| pregabalin | pregabalin | 1141200004 |
| lyrica 25mg capsule | pregabalin | 1141200072 |

# *Supplementary Material 9.* Type 2 diabetes ICD-9 and ICD-10 codes used to define cases in this study

|  | **UKB code** | **Diagnosis** |
| --- | --- | --- |
| **ICD-9 code** |  |  |
| 25000 | 25000 | Diabetes mellitus without mention of complication (adult-onset type) |
| 25010 | 25010 | Diabetes with ketoacidosis (adult-onset type) |
| 25020 | 25020 | Diabetes with coma (adult-onset type) |
| 25090 | 25090 | Diabetes with unspecified complications (adult-onset type) |
| **ICD-10 code** |  |  |
| *E11* | *E11* | *Non-insulin-dependent diabetes mellitus* |
| E11.0 | E110 | With coma |
| E11.1 | E111 | With ketoacidosis |
| E11.2 | E112 | With renal complications |
| E11.3 | E113 | With ophthalmic complications |
| E11.4 | E114 | With neurological complications |
| E11.5 | E115 | With peripheral circulatory complications |
| E11.6 | E116 | With other specified complications |
| E11.7 | E117 | With multiple complications |
| E11.8 | E118 | With unspecified complications |
| E11.9 | E119 | Without complications |

*Note.* Diagnoses adopted directly from International Statistical Classification of Diseases (ICD-9, ICD-10), as indicated on the UKB website.

# *Supplementary Material 10.* Other diabetes ICD-9 and ICD-10 codes used to exclude participants from cases and controls in this study

|  | **UKB code** | **Diagnosis** |
| --- | --- | --- |
| **ICD-9 code** |  |  |
| 25011 | 25011 | Diabetes with ketoacidosis (juvenile type) |
| 25021 | 25021 | Diabetes with coma (juvenile type) |
| 25091 | 25091 | Diabetes with unspecified complications (juvenile type) |
| 5881 | 5881 | Nephrogenic diabetes insipidus |
| 7751 | 7751 | Neonatal diabetes mellitus |
| **ICD-10 code** |  |  |
| *E10* | *E10* | *Insulin-dependent diabetes mellitus* |
| E10.0 | E100 | With coma |
| E10.1 | E101 | With ketoacidosis |
| E10.2 | E102 | With renal complications |
| E10.3 | E103 | With ophthalmic complications |
| E10.4 | E104 | With neurological complications |
| E10.5 | E105 | With peripheral circulatory complications |
| E10.6 | E106 | With other specified complications |
| E10.7 | E107 | With multiple complications |
| E10.8 | E108 | With unspecified complications |
| E10.9 | E109 | Without complications |
| *E12* | *E12* | *Malnutrition-related diabetes mellitus* |
| E12.0 | E120 | With coma |
| E12.1 | E121 | With ketoacidosis |
| E12.2 | E122 | With renal complications |
| E12.3 | E123 | With ophthalmic complications |
| E12.4 | E124 | With neurological complications |
| E12.5 | E125 | With peripheral circulatory complications |
| E12.6 | E126 | With other specified complications |
| E12.7 | E127 | With multiple complications |
| E12.8 | E128 | With unspecified complications |
| E12.9 | E129 | Without complications |
| *E13* | *E13* | *Other specified diabetes mellitus* |
| E13.0 | E130 | With coma |
| E13.1 | E131 | With ketoacidosis |
| E13.2 | E132 | With renal complications |
| E13.3 | E133 | With ophthalmic complications |
| E13.4 | E134 | With neurological complications |
| E13.5 | E135 | With peripheral circulatory complications |
| E13.6 | E136 | With other specified complications |
| E13.7 | E137 | With multiple complications |
| E13.8 | E138 | With unspecified complications |
| E13.9 | E139 | Without complications |
| *E14* | *E14* | *Unspecified diabetes mellitus* |
| E14.0 | E140 | With coma |
| E14.1 | E141 | With ketoacidosis |
| E14.2 | E142 | With renal complications |
| E14.3 | E143 | With ophthalmic complications |
| E14.4 | E144 | With neurological complications |
| E14.5 | E145 | With peripheral circulatory complications |
| E14.6 | E146 | With other specified complications |
| E14.7 | E147 | With multiple complications |
| E14.8 | E148 | With unspecified complications |
| E14.9 | E149 | Without complications |

Diagnoses adopted directly from International Statistical Classification of Diseases (ICD-9, ICD-10), as indicated on the UKB website.

# *Supplementary Material 11.* Coronary artery disease ICD-9 and ICD-10 codes used in this study

|  | **UKBB code** | **Diagnosis** |
| --- | --- | --- |
| **ICD-9 code** |  |  |
| 4109 | 4019 | Acute myocardial infarction |
| 4119 | 4119 | Other acute and subacute forms of ischaemic heart disease |
| 4129 | 4129 | Old myocardial infarction |
| 4139 | 4139 | Angina pectoris |
| 4140 | 4140 | Coronary atherosclerosis |
| 4141 | 4141 | Aneurysm of heart |
| 4148 | 4148 | Other specified forms of chronic ischaemic heart disease |
| 4149 | 4149 | Chronic ischaemic heart disease, unspecified |
| **ICD-10 code** |  |  |
| *I20* | *I20* | *Angina pectoris* |
| I20.0 | I200 | Unstable angina |
| I20.1 | I201 | Angina pectoris with documented spasm |
| I20.8 | I208 | Other forms of angina pectoris |
| I20.9 | I209 | Angina pectoris, unspecified |
| *I21* | *I21* | *Acute myocardial infarction* |
| I21.0 | I210 | Acute transmural myocardial infarction of anterior wall |
| I21.1 | I211 | Acute transmural myocardial infarction of inferior wall |
| I21.2 | I212 | Acute transmural myocardial infarction of other sites |
| I21.3 | I213 | Acute transmural myocardial infarction of unspecified site |
| I21.4 | I214 | Acute subendocardial myocardial infarction |
| I21.9 | I219 | Acute myocardial infarction, unspecified |
| *I22* | *I22* | *Subsequent myocardial infarction* |
| I22.0 | I220 | Subsequent myocardial infarction of anterior wall |
| I22.1 | I221 | Subsequent myocardial infarction of inferior wall |
| I22.8 | I228 | Subsequent myocardial infarction of other sites |
| I22.9 | I229 | Subsequent myocardial infarction of unspecified site |
| *I23* | *I23* | *Certain current complications following acute myocardial infarction* |
| I23.0 | I230 | Haemopericardium as current complication following acute myocardial infarction |
| I23.1 | I231 | Atrial septal defect as current complication following acute myocardial infarction |
| I23.2 | I232 | Ventricular septal defect as current complication following acute myocardial infarction |
| I23.3 | I233 | Rupture of cardiac wall without haemopericardium as current complication following acute myocardial infarction |
| I23.4 | I234 | Rupture of chordae tendineae as current complication following acute myocardial infarction |
| I23.5 | I235 | Rupture of papillary muscle as current complication following acute myocardial infarction |
| I23.6 | I236 | Thrombosis of atrium, auricular appendage and ventricle as current complications following acute myocardial infarction |
| I23.8 | I238 | Other current complications following acute myocardial infarction |
| *I24* | *I24* | *Other acute ischaemic heart diseases* |
| I24.0 | I240 | Coronary thrombosis not resulting in myocardial infarction |
| I24.1 | I241 | Dressler's syndrome |
| I24.8 | I248 | Other forms of acute ischaemic heart disease |
| I24.9 | I249 | Acute ischaemic heart disease, unspecified |
| *I25* | *I25* | *Chronic ischaemic heart disease* |
| I25.0 | I250 | Atherosclerotic cardiovascular disease, so described |
| I25.1 | I251 | Atherosclerotic heart disease |
| I25.2 | I252 | Old myocardial infarction |
| I25.3 | I253 | Aneurysm of heart |
| I25.4 | I254 | Coronary artery aneurysm |
| I25.5 | I255 | Ischaemic cardiomyopathy |
| I25.6 | I256 | Silent myocardial ischaemia |
| I25.8 | I258 | Other forms of chronic ischaemic heart disease |
| I25.9 | I259 | Chronic ischaemic heart disease, unspecified |

*Note.* Diagnoses adopted directly from International Statistical Classification of Diseases (ICD-9, ICD-10), as indicated on the UKB website.

# *Supplementary Material 12.* Operation codes used to define coronary artery disease

| **OPCS-4** **code** | **UKB code** | **Diagnosis** |
| --- | --- | --- |
| *K40* | *K40* | *Saphenous vein graft replacement of coronary artery* |
| K40.1 | K401 | Saphenous vein graft replacement of one coronary artery |
| K40.2 | K402 | Saphenous vein graft replacement of two coronary arteries |
| K40.3 | K403 | Saphenous vein graft replacement of three coronary arteries |
| K40.4 | K404 | Saphenous vein graft replacement of four or more coronary arteries |
| K40.8 | K408 | Other specified saphenous vein graft replacement of coronary artery |
| K40.9 | K409 | Unspecified saphenous vein graft replacement of coronary artery |
| *K41* | *K41* | *Other autograft replacement of coronary artery* |
| K41.1 | K411 | Autograft replacement of one coronary artery NEC |
| K41.2 | K412 | Autograft replacement of two coronary arteries NEC |
| K41.3 | K413 | Autograft replacement of three coronary arteries NEC |
| K41.4 | K414 | Autograft replacement of four or more coronary arteries NEC |
| K41.8 | K418 | Other specified other autograft replacement of coronary artery |
| K41.9 | K419 | Unspecified other autograft replacement of coronary artery |
| *K42* | *K42* | *Allograft replacement of coronary artery* |
| K42.1 | K421 | Allograft replacement of one coronary artery |
| K42.2 | K422 | Allograft replacement of two coronary arteries |
| K42.3 | K423 | Allograft replacement of three coronary arteries |
| K42.4 | K424 | Allograft replacement of four or more coronary arteries |
| K42.8 | K428 | Other specified allograft replacement of coronary artery |
| K42.9 | K429 | Unspecified allograft replacement of coronary artery |
| *K43* | *K43* | *Prosthetic replacement of coronary artery* |
| K43.1 | K431 | Prosthetic replacement of one coronary artery |
| K43.2 | K432 | Prosthetic replacement of two coronary arteries |
| K43.3 | K433 | Prosthetic replacement of three coronary arteries |
| K43.4 | K434 | Prosthetic replacement of four or more coronary arteries |
| K43.8 | K438 | Other specified prosthetic replacement of coronary artery |
| K43.9 | K439 | Unspecified prosthetic replacement of coronary artery |
| K44 | K44 | Other replacement of coronary artery |
| K44.1 | K441 | Replacement of coronary arteries using multiple methods |
| K44.2 | K442 | Revision of replacement of coronary artery |
| K44.8 | K448 | Other specified other replacement of coronary artery |
| K44.9 | K449 | Unspecified other replacement of coronary artery |
| *K45* | *K45* | *Connection of thoracic artery to coronary artery* |
| K45.1 | K451 | Double anastomosis of mammary arteries to coronary arteries |
| K45.2 | K452 | Double anastomosis of thoracic arteries to coronary arteries NEC |
| K45.3 | K453 | Anastomosis of mammary artery to left anterior descending coronary artery |
| K45.4 | K454 | Anastomosis of mammary artery to coronary artery NEC |
| K45.5 | K455 | Anastomosis of thoracic artery to coronary artery NEC |
| K45.6 | K456 | Revision of connection of thoracic artery to coronary artery |
| K45.8 | K458 | Other specified connection of thoracic artery to coronary artery |
| K45.9 | K459 | Unspecified connection of thoracic artery to coronary artery |
| *K46* | *K46* | *Other bypass of coronary artery* |
| K46.1 | K461 | Double implantation of mammary arteries into heart |
| K46.2 | K462 | Double implantation of thoracic arteries into heart NEC |
| K46.3 | K463 | Implantation of mammary artery into heart NEC |
| K46.4 | K464 | Implantation of thoracic artery into heart NEC |
| K46.5 | K465 | Revision of implantation of thoracic artery into heart |
| K46.8 | K468 | Other specified other bypass of coronary artery |
| K46.9 | K469 | Unspecified other bypass of coronary artery |
| *K50* | *K50* | *Other therapeutic transluminal operations on coronary artery* |
| K50.1 | K501 | Percutaneous transluminal laser coronary angioplasty |
| K75 | K75 | Percutaneous transluminal balloon angioplasty and insertion of stent into coronary artery |
| K75.1 | K751 | Percutaneous transluminal balloon angioplasty and insertion of 1-2 drug-eluting stents into coronary artery |
| K75.2 | K752 | Percutaneous transluminal balloon angioplasty and insertion of 3 or more drug-eluting stents into coronary artery |
| K75.3 | K753 | Percutaneous transluminal balloon angioplasty and insertion of 1-2 stents into coronary artery |
| K75.4 | K754 | Percutaneous transluminal balloon angioplasty and insertion of 3 or more stents into coronary artery NEC |
| K75.8 | K758 | Other specified percutaneous transluminal balloon angioplasty and insertion of stent into coronary artery |
| K75.9 | K759 | \|  \| Unspecified percutaneous transluminal balloon angioplasty and insertion of stent into coronary artery \| \| --- \| --- \| |

Surgery procedures adopted according to the Office of Population Censuses and Surveys Classification of Interventions and Procedures, version 4 (OPCS-4), directly from the UKB website.

# *Supplementary Material 13.* Availability of GWAS summary statistics used to create polygenic risk scores and calculate genetic correlations.

| **Trait** | **Consortium** | **Availability of the summary statistics/ download link** |
| --- | --- | --- |
| Bipolar disorder  (Stahl et al., 2019) | PGC | https://www.med.unc.edu/pgc/results-and-downloads/ |
| Systolic blood pressure (Evangelou et al., 2018) | ICBP ^a^ | Requested from authors |
| Diastolic blood pressure (Evangelou et al., 2018) | ICBP ^a^ | Requested from authors |
| Body mass index  (Locke et al., 2015) | GIANT | <https://portals.broadinstitute.org/collaboration/giant/index.php/GIANT_consortium_data_files> |
| Body mass index (Yengo et al., 2018) | GIANT ^b^ | <https://portals.broadinstitute.org/collaboration/giant/index.php/GIANT_consortium_data_files> |
| Waist-to-hip ratio (Shungin et al., 2015) | GIANT | http://portals.broadinstitute.org/collaboration/giant/images/5/54/GIANT_2015_WHR_COMBINED_EUR.txt.gz |
| Waist-to-hip ratio adjusted for BMI  (Shungin et al., 2015) | GIANT | http://portals.broadinstitute.org/collaboration/giant/images/e/eb/GIANT_2015_WHRadjBMI_COMBINED_EUR.txt.gz |
| HbA1c  (Wheeler et al., 2017) | MAGIC | ftp://ftp.sanger.ac.uk/pub/magic/HbA1c_METAL_European.txt.gz |
| Total Cholesterol  (Willer et al., 2013) | GLGC | http://csg.sph.umich.edu/willer/public/lipids2013/ |
| Triglycerides  (Willer et al., 2013) | GLGC | http://csg.sph.umich.edu/willer/public/lipids2013/ |
| High-density lipoprotein cholesterol  (Willer et al., 2013) | GLGC | http://csg.sph.umich.edu/willer/public/lipids2013/ |
| Coronary artery disease  (Nikpay et al., 2015) | CARDIoGRAM | [http://www.cardiogramplusc4d.org/data-downloads/](https://eur03.safelinks.protection.outlook.com/?url=http%3A%2F%2Fwww.cardiogramplusc4d.org%2Fdata-downloads%2F&data=01%7C01%7Canna.furtjes%40kcl.ac.uk%7C1c370f7704a74cd7b95008d6fa5050db%7C8370cf1416f34c16b83c724071654356%7C0&sdata=7B3GppdCeggDpzYHw5SKZZrGck0oHt2k7%2F2xQ7BZHsM%3D&reserved=0) |
| Stroke  (Malik et al., 2018) | MEGASTROKE | [http://www.megastroke.org](https://eur03.safelinks.protection.outlook.com/?url=http%3A%2F%2Fwww.megastroke.org&data=01%7C01%7Canna.furtjes%40kcl.ac.uk%7C1c370f7704a74cd7b95008d6fa5050db%7C8370cf1416f34c16b83c724071654356%7C0&sdata=w2eSa9KbxZLZhEaMlnPznbejeVGXQPvh%2FqZjqR%2FrDwI%3D&reserved=0) |
| Diabetes type 2  (Scott et al., 2017) | DIAGRAM | [http://diagram-consortium.org/downloads.html](https://eur03.safelinks.protection.outlook.com/?url=http%3A%2F%2Fdiagram-consortium.org%2Fdownloads.html&data=01%7C01%7Canna.furtjes%40kcl.ac.uk%7C1c370f7704a74cd7b95008d6fa5050db%7C8370cf1416f34c16b83c724071654356%7C0&sdata=SnNadv9dmneE4Klmm6WK01Jd%2FNHf9fZDNXmWNZvkyYE%3D&reserved=0) |

^a^ The blood pressure GWAS summary statistics contained the UKB study. This version was used for genetic correlations in this study. ICBP only summary statistics (excluding UKB) were used to create PRS to avoid overfitting. ^b^ The BMI (Yengo et al., 2018) GWAS summary statistics, which we used to calculate genetic correlations, were discovered in the UKB which is why we used summary statistics from (Locke et al., 2015) for PRS calculation.

Note. Hypertension was not included in the PRS analyses as, to our knowledge, there are no available GWAS summary statistics.

# *Supplementary Material 14.* Statistical models used for statistical analysis

| **Analysis** | **Model** |
| --- | --- |
| *Phenotypic analyses* | |
| Primary analysis, analyses for males and females separately, analyses limited to more severe BPD cases | BPD ~ CMT + assessment centre (+ fasting time for biomarkers) |
| *PRS associations* | |
| Primary analyses, analyses for males and females separately, analyses limited to more severe BPD cases | BPD ~ CMT PRS + PC1 + PC2 + PC3 + PC4 + PC5 + PC6 + assessment centre + batch |
| Sensitivity analyses for binary traits (excluding CAD and T2D cases) | BPD ~ CMT PRS + PC1 + PC2 + PC3 + PC4 + PC5 + PC6 + assessment centre + batch |
| Sensitivity analyses for continuous traits | BPD ~ CMT PRS + CMT + PC1 + PC2 + PC3 + PC4 + PC5 + PC6 + assessment centre + batch |

References

Abraham, G., Qiu, Y., & Inouye, M. (2017). Flashpca2: Principal Component Analysis of Biobank-Scale Genotype Datasets. *Bioinformatics, 33*, 2776–2778.

Bycroft, C., Freeman, C., Petkova, D., Band, G., Elliott, L. T., Sharp, K., . . . Marchini, J. (2017). Genome-Wide Genetic Data on ~500,000 Uk Biobank Participants. *bioRxiv*, 166298. doi:10.1101/166298

Cerimele, J. M., Chwastiak, L. A., Dodson, S., & Katon, W. J. (2014). The Prevalence of Bipolar Disorder in General Primary Care Samples: A Systematic Review. *General Hospital Psychiatry, 36*(1), 19-25. doi:10.1016/j.genhosppsych.2013.09.008

Coleman, J. R. I., Peyrot, W. J., Purves, K. L., Davis, K. A. S., Rayner, C., Choi, S. W., . . . Breen, G. (2019). Genome-Wide Gene-Environment Analyses of Major Depressive Disorder and Reported Lifetime Traumatic Experiences in Uk Biobank. *bioRxiv*, 247353. doi:10.1101/247353

Davis, K. A. S., Coleman, J. R. I., Adams, M., Allen, N., Breen, G., Cullen, B., . . . Hotopf, M. (2020). Mental Health in Uk Biobank – Development, Implementation and Results from an Online Questionnaire Completed by 157 366 Participants: A Reanalysis. *BJPsych Open, 6*(2), e18. doi:10.1192/bjo.2019.100

Evangelou, E., Warren, H. R., Mosen-Ansorena, D., Mifsud, B., Pazoki, R., Gao, H., . . . the Million Veteran, P. (2018). Genetic Analysis of over 1 Million People Identifies 535 New Loci Associated with Blood Pressure Traits. *Nature Genetics, 50*(10), 1412-1425. doi:10.1038/s41588-018-0205-x

Locke, A. E., Kahali, B., Berndt, S. I., Justice, A. E., Pers, T. H., Day, F. R., . . . Speliotes, E. K. (2015). Genetic Studies of Body Mass Index Yield New Insights for Obesity Biology. *Nature, 518*, 197–206. doi:10.1038/nature14177

Manichaikul, A., Mychaleckyj, J. C., Rich, S. S., Daly, K., Sale, M., & Chen, W.-M. (2010). Robust Relationship Inference in Genome-Wide Association Studies. *Bioinformatics, 26*(22), 2867-2873. doi:10.1093/bioinformatics/btq559

Nikpay, M., Goel, A., Won, H.-H., Hall, L. M., Willenborg, C., Kanoni, S., . . . Consortium, t. C. D. (2015). A Comprehensive 1000 Genomes–Based Genome-Wide Association Meta-Analysis of Coronary Artery Disease. *Nature Genetics, 47*, 1121. doi:10.1038/ng.3396

Pini, S., de Queiroz, V., Pagnin, D., Pezawas, L., Angst, J., Cassano, G. B., & Wittchen, H.-U. (2005). Prevalence and Burden of Bipolar Disorders in European Countries. *European Neuropsychopharmacology, 15*(4), 425-434. doi:10.1016/j.euroneuro.2005.04.011

Schnier, C., Bush, K., Nolan, J., & Sudlow, C., on behalf of UK Biobank Outcome Adjudication Group,. (2017). Definitions of Stroke for Uk Biobank Phase 1 Outcomes Adjudication. Retrieved from biobank.ndph.ox.ac.uk/showcase/docs/alg_outcome_stroke.pdf

Scott, R. A., Scott, L. J., Mägi, R., Marullo, L., Gaulton, K. J., Kaakinen, M., . . . Prokopenko, I. (2017). An Expanded Genome-Wide Association Study of Type 2 Diabetes in Europeans. *Diabetes, 66*(11), 2888-2902. doi:10.2337/db16-1253

Shungin, D., Winkler, T. W., Croteau-Chonka, D. C., Ferreira, T., Locke, A. E., Mägi, R., . . . Mohlke, K. L. (2015). New Genetic Loci Link Adipose and Insulin Biology to Body Fat Distribution. *Nature, 518*, 187. doi:10.1038/nature14132

Smith, D. J., Nicholl, B. I., Cullen, B., Martin, D., Ul-Haq, Z., Evans, J., . . . Pell, J. P. (2013). Prevalence and Characteristics of Probable Major Depression and Bipolar Disorder within Uk Biobank: Cross-Sectional Study of 172,751 Participants. *PLOS ONE, 8*(11), e75362. doi:10.1371/journal.pone.0075362

Stahl, E. A., Breen, G., Forstner, A. J., McQuillin, A., Ripke, S., Trubetskoy, V., . . . the Bipolar Disorder Working Group of the Psychiatric Genomics, C. (2019). Genome-Wide Association Study Identifies 30 Loci Associated with Bipolar Disorder. *Nature Genetics, 51*(5), 793-803. doi:10.1038/s41588-019-0397-8

Sudlow, C., Gallacher, J., Allen, N., Beral, V., Burton, P., Danesh, J., . . . Collins, R. (2015). Uk Biobank: An Open Access Resource for Identifying the Causes of a Wide Range of Complex Diseases of Middle and Old Age. *PLOS Medicine, 12*(3), e1001779. doi:10.1371/journal.pmed.1001779

Warren, H. R., Evangelou, E., Cabrera, C. P., Gao, H., Ren, M., Mifsud, B., . . . Elliott, P. (2017). Genome-Wide Association Analysis Identifies Novel Blood Pressure Loci and Offers Biological Insights into Cardiovascular Risk. *Nature Genetics, 49*, 403. doi:10.1038/ng.3768

Wheeler, E., Leong, A., Liu, C.-T., Hivert, M.-F., Strawbridge, R. J., Podmore, C., . . . Meigs, J. B. (2017). Impact of Common Genetic Determinants of Hemoglobin A1c on Type 2 Diabetes Risk and Diagnosis in Ancestrally Diverse Populations: A Transethnic Genome-Wide Meta-Analysis. *PLOS Medicine, 14*(9), e1002383. doi:10.1371/journal.pmed.1002383

Willer, C. J., Schmidt, E. M., Sengupta, S., Peloso, G. M., Gustafsson, S., Kanoni, S., . . . Global Lipids Genetics Consortium. (2013). Discovery and Refinement of Loci Associated with Lipid Levels. *Nature Genetics, 45*, 1274. doi:10.1038/ng.2797

World Health Organization. (2004). International Statistical Classification of Diseases and Related Health Problems (10th Revision). Retrieved from <https://icd.who.int/browse10/2016/en>

Yengo, L., Sidorenko, J., Kemper, K. E., Zheng, Z., Wood, A. R., Weedon, M. N., . . . Consortium, t. G. (2018). Meta-Analysis of Genome-Wide Association Studies for Height and Body Mass Index in ∼700000 Individuals of European Ancestry. *Human Molecular Genetics, 27*(20), 3641-3649. doi:10.1093/hmg/ddy271
